# Supplementary figures and images for: Dual Inhibition of HIF-1α and HIF-2α as a Promising Treatment for VHL-Associated Hemangioblastomas: A Pilot Study Using Patient-Derived Primary Cell Cultures
Source: Biomedicines. 2025 May 19;13(5):1234. doi: 10.3390/biomedicines13051234 (PMC12108798; doi:10.3390/biomedicines13051234)

Supplementary Figure 1 (Figure S1)

UNCROPPED WESTERN-BLOTS for Figure 4b

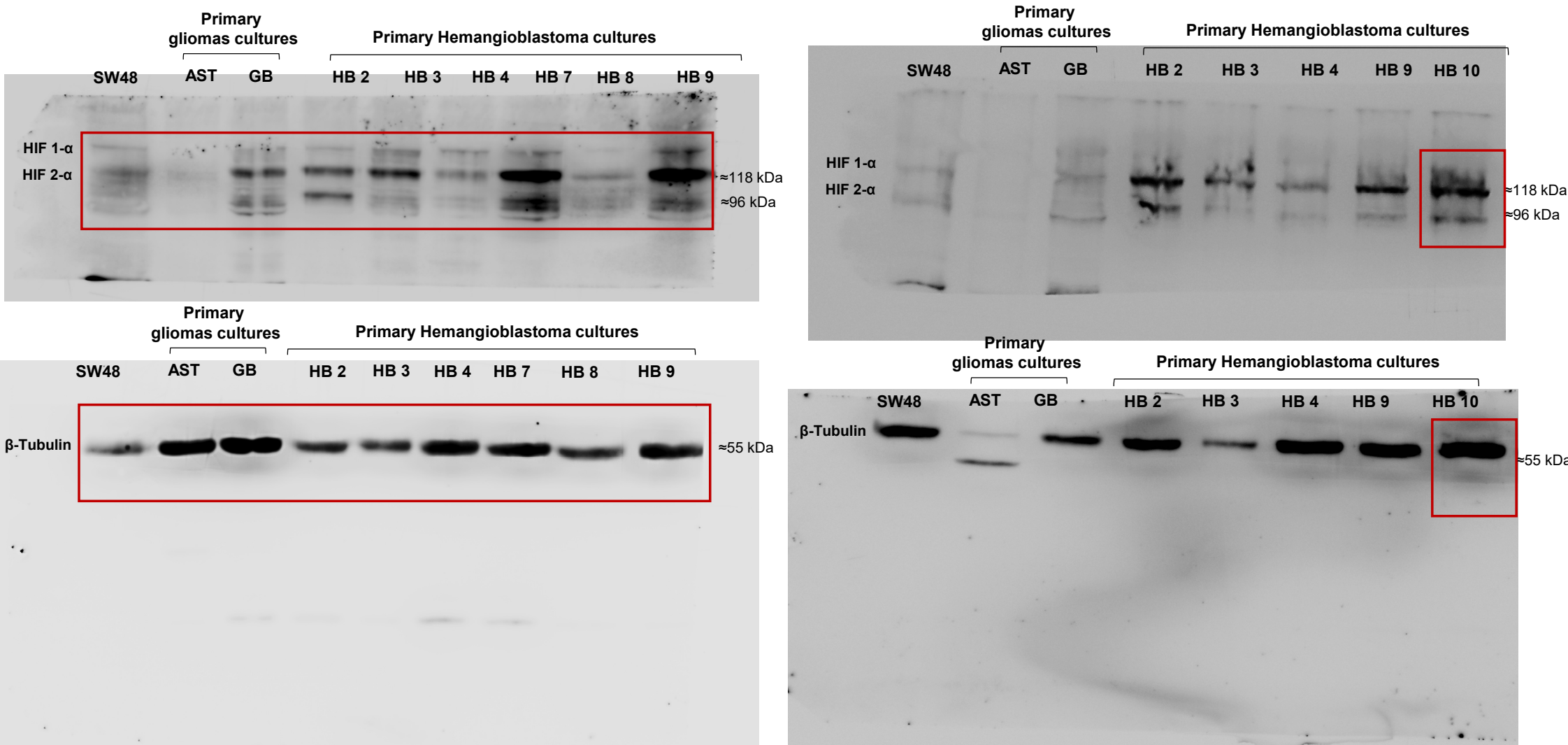

Supplement: Supplementary file 1 [file biomedicines-13-01234-s001.zip › biomedicines-3560244-Supplementary Figure, S1.pdf]
